# Diverse residents’ experiences of policing and attitudes towards law enforcement: Findings from a large community-based survey in San José, California

**DOI:** 10.1371/journal.pone.0325257

**Published:** 2025-05-29

**Authors:** Soma de Bourbon, Michael Dao, Melissa McClure Fuller, William Armaline, Curtis Asplund, Rachel Kumar, Daniel DeSanto, Jaala Robinson, Derrick Sanderlin, Miranda Worthen

**Affiliations:** 1 Department of Sociology and Interdisciplinary Social Sciences, San José State University, San José, California, United States of America; 2 Department of Kinesiology, San José State University, San José, California, United States of America; 3 Department of Public Health, San José State University, San José, California, United States of America; 4 Department of Physics & Astronomy, San José State University, San José, California, United States of America; 5 Sacred Heart Community Service, San José, California, United States of America; Hunan University, CHINA

## Abstract

Despite widespread debate on public safety policy, few studies explore diverse community members’ perspectives on safety and policing. This community-based participatory research study assessed perspectives on city spending, opinions about law enforcement policy, perspectives on police and safety, direct experiences with police, and perspectives on alternatives to policing in San José, California, one of the ten largest cities in the United States. Between July 27, 2021 – January 14, 2022, we conducted an online survey, available in 7 languages, and obtained 1,595 responses. Respondents supported increased funding for community safety resources (73%) and helping residents meet basic needs (67%). A majority of respondents felt policing in San José had serious problems, requiring major reform. Across a range of questions, sexual and gender minorities, younger people, African American/Black, Native American, Chicanx/Latinx/Hispanic respondents, and people with lower incomes had more negative attitudes towards and experiences with police compared to those who identify as men, heterosexual, older, White or Asian, or had higher incomes. There was strong support (72% − 82%) for four alternative-to-police policy proposals. As communities across the country grapple with how to achieve safety, especially for residents who are disproportionately harmed by police, community-based participatory research is a valuable tool for engaging, understanding, and mobilizing communities.

## Introduction

The summer of 2020 saw a nationwide movement calling for justice for victims of police brutality and racial inequality, sparked by the killing of George Floyd in Minneapolis, Minnesota [1,2]. The Black Lives Matter movement gained renewed momentum, with protests and demonstrations taking place across the country and around the world [3]. Alongside these demonstrations, there emerged a public conversation about shifting funding toward social programs and community-based alternatives to law enforcement and away from policing [4]. Indeed, with police violence ranking sixth in the leading causes of death for young Black men, “public health-informed alternative responses programs” have emerged as a strategy to prevent harm, especially to marginalized community members, and to reduce health disparities [5,6]. The call for alternative response programs has frequently been met with resistance, with politicians and community leaders arguing that reducing police funding jeopardizes public safety, and many cities increased funding for law enforcement in the aftermath of the Black Lives Matter protests [7,8].

The contours of the debate about police funding and alternatives to policing are both ideological and pragmatic, and are influenced by the national conversation, while also being highly contextual and local. While the debate about how to best achieve community safety remains polarized, parts of the country have experimented with developing alternative non-police responses, especially for mental health crises, and other communities have expanded police budgets and scope of work [9,10].

Despite this growing national conversation around police spending and community safety, there is a notable gap in the literature reporting on studies that elicit the opinions of highly diverse residents of local communities on these issues. One exception is a 2022 statewide opinion poll of California voters, which found that 34% favored adopting alternative response programs without police and disaggregated the response by political party, age, race, and income [11]. In this survey, non-police alternative response programs had the strongest support from those with no party preference, those over age 50, American Indian and White voters, and people with incomes between $60,000 - $199,999 [11].

Within the criminal justice literature, there has been extensive research on satisfaction with police, as this is conceived of as critical to the success of community policing strategies [12]. Although “racial background” is a consistent predictor of attitude towards police, the operationalization of race in most studies is extremely limited [13,14]. Most studies focus on narrowly defined demographic groups, comparing perspectives of Black and White participants without considering experiences of other racialized groups [14].

There have also been few studies that examine how other aspects of identity may shape individuals’ experiences and perspectives, such as sexual orientation, gender identity, socioeconomic status, or nativity [14]. A notable exception is a small study that employed an intersectional approach to examine differences in satisfaction with police by race, gender, and class [12]. While the study found a higher level of satisfaction among participants who were White, male, and higher income than other participants, the study included fewer than 150 participants and thus inference was limited [12]. Two additional studies that have examined more nuanced differences in experiences and perspectives by identity but have focused on specific sub-populations: “Policing the Rainbow” explored the experiences of lesbian, gay, bisexual, transgender, queer, or questioning (LGBTQ+) individuals throughout the United States, and the “Futuro Y Esperanza” study examined Latinx perspectives on policing and safety [15,16]. Both studies found significant within-group differences based on other aspects of identity, such as race, gender, and socioeconomic status [15,16].

There is a lack of understanding of where similarities and differences may exist among diverse residents. Aside from attitudes towards police, how do community members believe that safety can be achieved? Are there specific non-police strategies that have broad support among diverse community members? How do diverse residents experience the police? Moreover, the concept of diversity is multifaceted and extends beyond narrow categories of race and ethnicity and includes factors such as gender, sexual orientation, and socio-economic status. No studies that we are aware of have taken a comprehensive approach to understanding diversity and examining how it intersects with residents’ perspectives on police spending and community safety. Such studies are crucial to informing policies and practice that are more inclusive and responsive to the needs of diverse communities.

The present study employed a community-based participatory approach to broadly understand diverse residents’ experiences with law enforcement and attitudes towards the police and community safety in San José, California.

## Local context

San José is demographically distinct from many of the other cities where social movements for reducing police funding emerged; cities like Minneapolis, Nashville, Los Angeles, and New York [7,17,18]. San José is the tenth largest city in the United States, with a population of just over a million [19]. The percentage of the city’s population that is foreign-born, at 38.9%, is higher than any of the other ten largest cities [19]. Among those born outside of the United States, 25% were born in Mexico, 21% were born in Vietnam, 11% were born in India, and 9% were born in China [19].

The racial and ethnic profile of San José is distinct from most other places in the United States. A third of the population identifies as Asian and a quarter each identify as White or Hispanic. Just under 3% of the population identifies as Black [20]. Compared to other large cities, San José has more Asian and fewer African American and White residents [19].

While the median household income is $115,000, there is stark inequality in income and wealth. Since 2012, income inequality in the region has grown twice as fast as in the rest of California or the United States; the median individual income for those with a bachelor’s degree or higher was $108,300, compared to only $31,700 for those without a high school degree [21,22]. While the average wealth of the top 10% of households in Silicon Valley is $5.8 million, 46% of children in Silicon Valley live in households that receive government anti-poverty assistance [21].

The People’s Budget of San José Study was a community-based participatory research study aimed at understanding the perspectives of diverse residents of San José, California on all aspects of policing and city spending. Community-based participatory research is an approach to research that seeks equitable collaboration by academic and community partners during all phases of a study: from the initial spark of an idea, to study design, data collection, analysis, and sharing the study results [23]. Community-based participatory research approaches build on the strengths and resources of community members, and they value lived expertise in addition to academic expertise [24]. In the present article, we focus on the results of a survey of San José residents that was conducted as part of the broader People’s Budget of San José Study.

The partners in this endeavor were the San José State University Human Rights Institute (HRI) and Sacred Heart Community Service. The HRI is a multi-disciplinary institute focusing on human rights praxis. HRI researchers contributed to study design, implementation, analysis, and dissemination. Sacred Heart is the largest direct service provider in the region. In addition to direct service provision, Sacred Heart has a community organizing mission aimed at engaging and strengthening the community to develop durable solutions to poverty. The Race, Equity, and Community Safety (RECS) Committee of Sacred Heart, a network of multi-racial community members working together to reimagine community safety, contributed to the design, implementation, analysis, and dissemination of the study. The Race, Equity, Action, and Leadership (REAL) Coalition, a network of leaders from a broad spectrum of over 80 community-based organizations dedicated to advancing racial justice in Silicon Valley, also contributed to the design and implementation of the study.

As an academic partner in this study, the HRI wanted to better understand the range of perspectives of San José residents, in light of their unique diversity. As a community organizing partner, Sacred Heart endorsed this exploratory objective, but also viewed the study itself as a way to promote conversation about policing and spending and to support coalition building. While this particular study began in the fall of 2020, the local context for this inquiry is important to understand.

Since 2001, Silicon Valley De-Bug, a community organizing, advocacy, and storytelling organization, had been working on criminal justice reform, immigrant rights, police accountability, racial justice, and economic justice, centering the perspectives of those impacted by police and the criminal justice system. While the national Black Lives Matter movement protests in the summer of 2020 were catalyzed by the killings of Breonna Taylor and George Floyd, the local movement was spurred on by the in-custody death of Michael Tyree in 2015, which led to three hunger strikes organized by people in the county jail and their families [25]. In August 2020, Silicon Valley De-Bug released a report putting forth specific recommendations for shifting the county budget towards community-based alternatives and away from policing and law enforcement [26].

Inspired by the uprising of support for Black Lives Matter in the summer of 2020, Sacred Heart and the Silicon Valley Council of Nonprofits brought together over 80 different local non-profit leaders to form the REAL Coalition [27–29]. The purpose of this coalition was to have a weekly meeting space to build support for racial justice. While San José witnessed very large, diverse, and sustained Black Lives Matter protests in the summer of 2020, no systematic inquiry had examined the extent of support for the movement’s demands among residents. Members of the REAL Coalition, who represent much of the diversity of the city, had a wide range of opinions about the level of support among city residents for pursuing non-police alternatives to promote public safety. One of the aims for this study was to help organizations better understand the opinions, ideas, and experiences of the people they serve. In addition, the San José City Council, like many other jurisdictions, began the process of convening an advisory committee to “re-imagine public safety” and it was believed that high quality research examining residents’ perspectives, experiences, and attitudes would help inform that entity’s deliberation process [30,31].

In this article, we describe both the process of this community-based participatory research study, as well as the results of the survey. While the specific study findings may not be generalizable to populations beyond San José, we believe the approach we used to better understand this diverse community’s perspectives is replicable to other communities. In addition, we believe this study adds significant nuance to the frequently polarized discourse about policing, by examining experiences and perspectives along several facets of diversity, and exploring where broad agreement exists in spite of differences.

## Methods

Academic and community partners began meeting in the Fall of 2020 to plan a mixed-methods research study to better understand San José residents’ perspectives on community safety, the city budget, and policing. We began by conducting a series of focus groups to get a sense of the range of ways that community members were thinking and speaking about these topics. HRI and Sacred Heart partners collaboratively designed a focus group discussion guide, organizational partners in the REAL coalition were recruited to conduct focus groups with the residents they serve, and an interdisciplinary group of researchers at the HRI analyzed the data from the focus groups and shared results back to the community through a public presentation, report, and, later, a journal article [32,33].

Building on what we learned in the focus groups, HRI research partners drafted a survey assessing residents’ perspectives on San José city spending, opinions about policing and law enforcement policy, experiences of policing, and perspectives on alternatives to policing. The draft survey also asked several demographic questions to characterize the population taking the survey and explore differences in responses by identity. We workshopped the survey draft during a meeting of the REAL coalition where members read through the draft survey, discussed it in small groups, and provided suggestions on constructs to add, remove, and modify. For example, one of the recommendations by the REAL coalition was to ask participant perspectives on specific alternatives to policing that were being considered by San José, including (1) the development of a 911 dispatched non-police mental health crisis team, (2) automated traffic enforcement tools to substitute for police traffic stops, (3) non-law enforcement response to people experiencing homelessness, and (4) replacing police school resource offices with trained counselors and coaches in San José schools (see S1 Table).

HRI research partners revised the survey and then engaged in a series of meetings with members of the Race, Equity, and Community Safety (RECS) committee of Sacred Heart to pilot test the survey and to develop appropriate data safety protocols. HRI and RECS established a joint Community Advisory Board Data Access Committee to guide decision-making regarding what analyses would be conducted, how these analyses would be presented, and whether and how data would be shared.

Once the survey was finalized, HRI recruited six undergraduate research interns who were members of different linguistic communities in San José. These interns worked with a partner to translate the survey from English into the most commonly spoken languages in San José: Amharic, Chinese, Farsi, Spanish, Tagalog, and Vietnamese. In order to ensure continuity of meaning, each survey was then back translated into English by a person who had not participated in the original translation. Each back translated survey was examined and any inconsistencies with the original English version were reviewed, discussed, and refined. Separate surveys were built in Qualtrics for each language and a single website was built where links to the survey in each of the seven languages were available. The study protocol was approved by the San José State University Institutional Review Board (Protocol #21153). We received a waiver of signed consent as the data were collected and analyzed anonymously.

**Fig 1:**
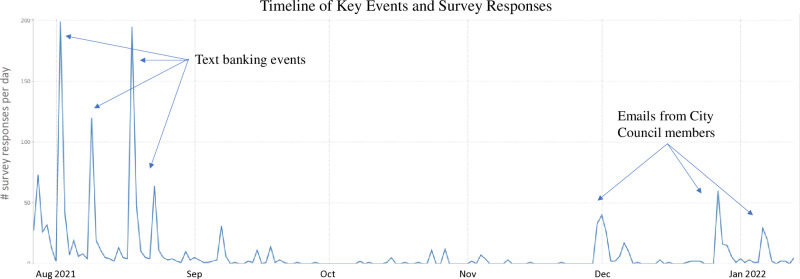
Timeline of Key Events and Survey Responses.

The survey was open from July 27, 2021 to January 14, 2022, with the bulk of responses collected in the first two months. During these months, REAL coalition partners distributed an invitation to take the survey to their membership email lists and via social media. RECS and HRI members solicited residents in person, including at citywide music festivals and other events. In addition, HRI interns and RECS members held four mass text banking events using voter registration rolls, texting over 64,000 registered voters in San José with an invitation to take the survey. In the final phase of the survey, HRI analyzed survey responses by participant reported zip code, calculating the proportion of residents in the zip code who had completed the survey, clustering zip codes by City Council district, and reaching out to City Council members where a lower proportion of residents had completed the survey than average. We asked these Council members to send an invitation to their constituents through various channels including email and social media encouraging them to complete the survey. We provide a detailed timeline of key events and survey responses in Fig 1. The largest concentration of survey responses (over half of all survey responses) were obtained within 12 hours of mass text banking events, suggesting that this solicitation strategy yielded the majority of responses, though this activity had a very low response rate (around 1–2%). Democrats represent 49.8% of registered voters in the county, compared to 46.2% of the state [34]. We also documented a surge in responses after the City Council members representing districts with lower participation emailed their constituents. While the study sample is a convenience sample, the population closely mirrors the population of San José along major demographic factors and has representation from all areas of the city.

The full survey is included in S1 Table. The introductory statement explaining the purpose of the survey noted that the survey was for community members who live, work, or study in San José. The survey asked participants whether they lived in San José and for their zip code. Participants were then given a series of statements about perspectives and experiences with safety and policing and asked their level of agreement, and to select from a range of statements the one that most closely matched their opinion. The survey then described four proposed policies to shift responsibility and/or funding away from the police to civilian agencies and asked people their level of support for or opposition to each of these policy proposals. A series of questions then asked participants their perspective on whether the city should spend more, the same, or less money on four categories of spending and provided an open-ended response for people’s comments on the overall city budget. Respondents were then asked if they had any experiences with police or law enforcement in the past five years and if they selected yes, they were asked whether the experience was generally positive, negative, or mixed. Finally, participants were asked a series of demographic questions including their age, gender, sexual orientation, race or ethnicity, level of education, household income, employment status, nativity, languages spoken in the household, and whether a household member had spent time in jail, prison, or immigration detention in the past six months and whether the participant was currently experiencing homelessness.

The survey data were extracted from Qualtrics, combined across the different language versions into a single spreadsheet, and imported into Stata version 17 where data were cleaned and analyzed. After initial descriptive analyses, the HRI research team met with RECS members in the Community Advisory Board Data Access Committee to share findings and identify which additional analyses to conduct, including how to analyze and present differences by demographic characteristics.

The Community Advisory Board Data Access Committee decided to focus our results on descriptive analyses, including examining survey items by demographic characteristics, but to refrain from directly comparing one group to another along single axes of identity. For questions regarding different experiences of policing, we decided to conduct an intersectional analysis to examine the differences in experience among those occupying multiple marginalized intersectional identities to those who occupy social positions with greater power. Intersectionality is a conceptual framework that infuses complexity into our understanding of identity, recognizing that inhabiting a position at the nexus of multiple inequities (e.g., race/ethnicity, sexual orientation, gender, and income status) can compound the negative effects of marginalization [35]. Specifically, we used an interaction term in a Poisson regression model with robust standard errors to estimate the likelihood of different experiences comparing participants who are white, heterosexual, cisgender men or women, with household incomes greater than $100,000 to participants who are non-white, gender or sexual minority, with household incomes under $100,000. This yields an estimate of the prevalence ratio comparing these two groups [36].

## Results

### Sample characteristics

There were 1,595 survey responses, with 92% of respondents taking the survey in English. 91% of respondents reported that they live in San José with participation distributed across the city’s zip codes. Demographic characteristics of the survey participants are summarized in Table 1. Participants represented a wide range of ages, genders, sexual orientations, and educational attainments and broadly reflected the racial and ethnic diversity of the city. Just under 5% of respondents reported that they or a member of their household had spent time in jail, prison, or immigration detention in the previous 6 months and 2% reported currently experiencing homelessness.

**Table 1:** Demographic Characteristics of Survey Participants.

Characteristic	Number, %
**Total**	1,595 (100)
**Age**	
12–17	13 (1)
18–25	253 (21)
26–39	340 (29)
40–64	465 (39)
65 and older	118 (10)
**Gender**	
Man	442 (37)
Non-Binary/Gender Non-Conforming	25 (2)
Woman	697 (59)
Transgender, Two-Spirit^a^, or Another Gender Identity^b^	21 (2)
**Sexual Orientation**	
Bisexual	86 (7)
Gay	33 (3)
Lesbian	13 (1)
Queer	28 (2)
Straight	962 (83)
Two-Spirit^a^ or Another Sexual Orientation	40 (3)
**Educational Attainment**	
Less Than High School	32 (3)
High School	220 (19)
Associate’s Degree	212 (18)
Bachelor’s Degree	398 (34)
Graduate or Professional Degree	283 (24)
Other	38 (3)
**Race and Ethnicity** ^c^	
African American or Black	140 (10)
Asian Indian	45 (3)
Chinese	57 (4)
Filipino	85 (6)
Vietnamese	106 (8)
Japanese, Korean, or Other Asian^b^	40 (4)
Chicano/a, Mexican American, or Mexican	191 (14)
Hispanic or Latinx	188 (13)
Middle Eastern	35 (2)
Native American or Indigenous	32 (2)
Pacific Islander or Native Hawaiian	17 (1)
White	409 (29)
Other Race or Ethnicity	57 (4)
**Born in the United States**	
Yes	860 (72)
No	307 (26)
**Household Income**	
$0 - $24,999	160 (14)
$25,000 - $49,000	187 (16)
$50,000 - $99,999	324 (28)
$100,000 - $199,999	302 (26)
$200,000 or more	177 (15)
**Employment Status**	
Full Time	595 (50)
Part Time	205 (17)
Student	131 (11)
Retired	111 (9)
Unemployed	78 (7)
Disabled	25 (2)
Other	38 (3)

^a^Two-Spirit is a term used by many Indigenous North Americans to describe people who represent a third and fourth gender and can refer to both a gender identity as well as a sexual orientation.

^b^Categories where there were fewer than 13 respondents are combined in this table to protect anonymity.

^c^Respondents were provided 14 non-mutually exclusive racial/ethnic categories to choose from, resulting in over 80 unique racial/ethnic identities. In this table, the 15% of respondents who selected more than one category are represented in each of the categories they selected, thus the number of responses is more than the total. For example, there were 34 participants who identified as African American and Chicano/a, Mexican American, or Mexican and these participants are counted in both categories.

Although comparisons with the Census are not straightforward because of the different categories used in the Census and the survey, using the Census as a comparator, there was slight overrepresentation of participants who identified as African American or Black (Survey 10% vs. Census 3%) and White (Survey 29% vs. Census 26%) and underrepresentation of Asian participants (Survey 26% vs. Census 36%) and Latino participants (Survey 27% vs. Census 32%). Survey respondents were more likely to have been born in the United States (Survey 72% vs. Census 60%) [20]. The overall distribution of household income among survey respondents was lower than the general population of San José ($0 - $24,999 Survey 14% vs. Census 9%; $25,000 - $49,999 Survey 16% vs. Census 10%; $50,000 - $99,99 Survey 28% vs. Census 21%; $100,000 - $199,999 Survey 26% vs. Census 31%; $200,000 + Survey 15% vs. Census 28%) [20].

### Perspectives on San José city spending

**Fig 2:**
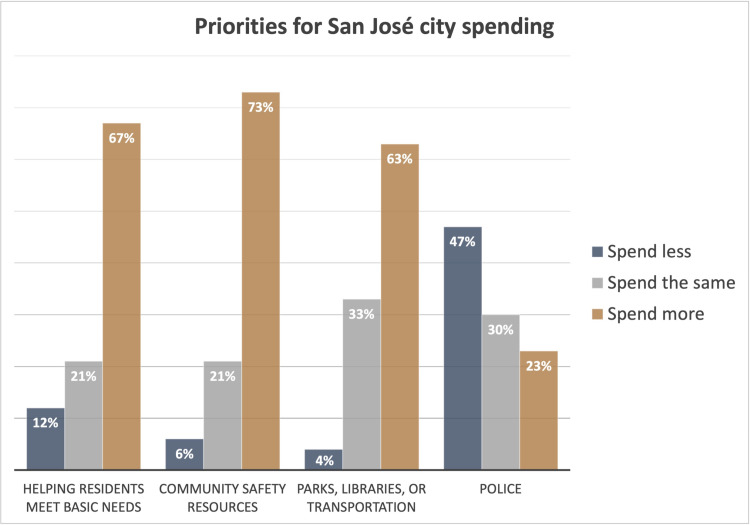
Priorities for San José city spending.

A robust majority of respondents supported increased funding for community safety resources (73%), helping residents meet basic needs (67%), and public resources like parks, libraries, and transportation (63%) (Fig 2). The area with the most support for decreased funding was police (47%). There were few differences by demographic groups in whether to spend more, the same, or less money on helping residents meet basic needs, community safety resources, and public resources like parks, libraries, and transportation. While there were some differences by demographic characteristics on police spending, there were no demographic categories where a majority of participants supported an increase in spending on policing. Relatively more support for increased spending on policing was observed among older participants compared to younger, men compared to women and gender minorities, heterosexual participants compared to all other sexual orientations, those born outside of the United States compared to those born in the United States, and those with household incomes higher than $100,000 compared to those with lower incomes.

In addition to the closed-ended questions on spending priorities, the survey included an opportunity to write in responses to the question “Do you have any suggestions about what San José’s budget priorities should be?” Over 600 participants made written comments expressing their thoughts on the city budget. We reviewed each comment and classified them by topic. The most common topics discussed in these open-ended comments were homelessness (mentioned in 30% of comments), the high cost of housing (mentioned in 23% of comments), mental health services (mentioned in 16% of comments), and education (mentioned in 13% of comments) (Table 2). Topics receiving fewer than 10% of the comments included infrastructure, public services, and safety.

**Table 2:** Suggested areas for San José spending and example quotes.

Topic	Example Quotes
Homelessness	*“Help/support for the homeless population should be a top priority.”* *“Homelessness needs to be addressed! It was not this bad even five years ago!”*
Housing	*“Affordable housing should have been the #1 priority a decade ago.”* *“Housing for middle class. As a single father of two young children making 100k per year, I cannot afford to buy a home.”*
Mental Health	*“Increase mental health funding. It’s extremely difficult for people to get help.”* *“Increase support for mental health issues.”*
Education	*“I believe San Jose should focus on making better schools for the youth.”* *“Raise the salaries of teachers and professors.”*

### Opinions about policing and law enforcement policy

**Fig 3:**
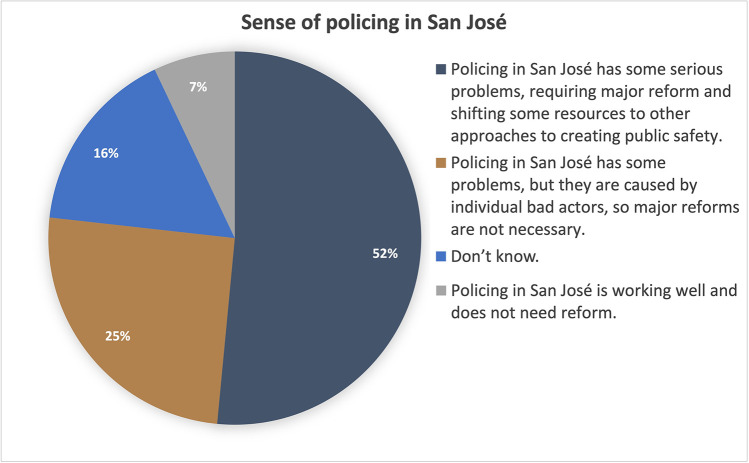
Sense of policing in San José.

**Fig 4:**
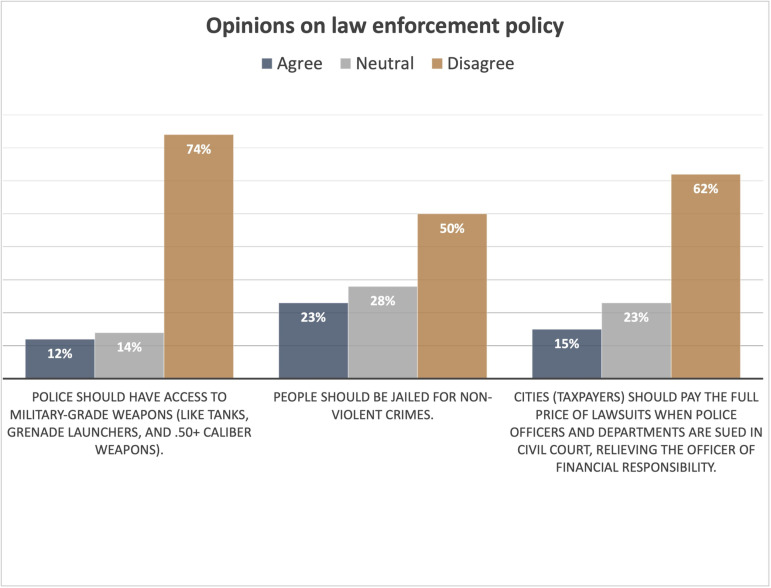
Opinions on law enforcement policy.

When asked about their overall perception of policing in San José, 52% of respondents agreed with the statement “policing in San José has some serious problems, requiring major reform and shifting some resources to other approaches to creating public safety” (Fig 3). Another 25% of respondents agreed with the statement “San José has some problems, but they are caused by individual bad actors, so major reforms are not necessary.” Of the remaining participants, 7% agreed with the statement “Policing in San José is working well and does not need reform” and 16% said they did not know. The survey also assessed participants’ attitudes towards three specific law enforcement policies (Fig 4). Most participants did not want police to have access to military-grade weapons, did not agree that people should be jailed for non-violent crimes, and did not want cities to pay for lawsuits for police misconduct.

### Perspectives on Police and Safety

**Fig 5:**
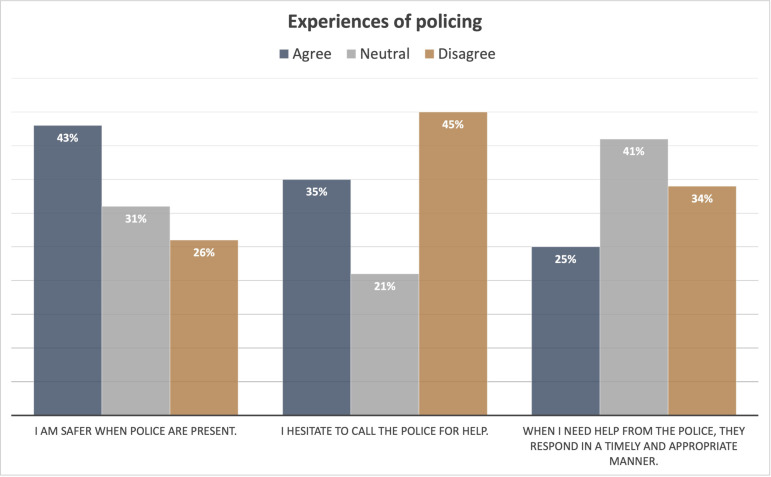
Experiences of policing.

The survey asked several questions aimed at understanding how safe respondents feel around the police and how they view police responsiveness. Participant perspectives on safety and police responsiveness varied widely based on their demographic groups. Across a range of questions, sexual and gender minorities, younger people, African American/Black, Native American, Chicanx/Latinx/Hispanic respondents, and people with lower household incomes had generally more negative assessment of the safety and responsiveness of police compared to those who identify as men, heterosexual, older, White or Asian, or had higher household incomes. Participants were asked whether they agreed, were neutral or undecided, or disagreed with several statements (Fig 5). In response to the statement “I am safer when police are present,” 43% of participants agreed, 31% were neutral, and 26% disagreed. In response to the statement “I hesitate to call the police for help,” 35% agreed, 21% were neutral, and 45% disagreed. In response to the statement “When I need help from the police, they respond in a timely and appropriate manner,” 25% of participants agreed, 41% were neutral, and 34% disagreed.

**Fig 6:**
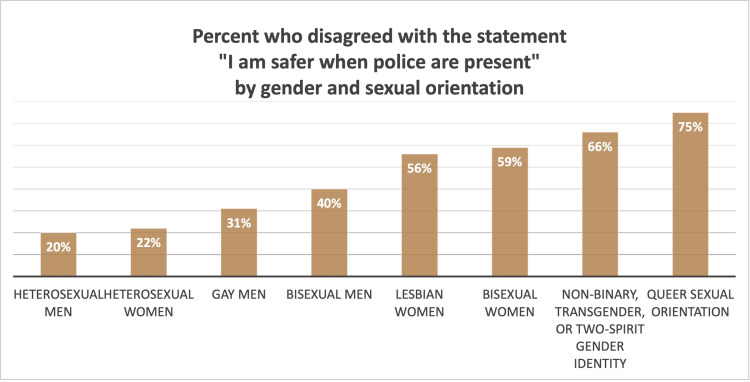
Percent who disagreed with the statement “I am safer when police are present” by gender and sexual orientation.

**Fig 7:**
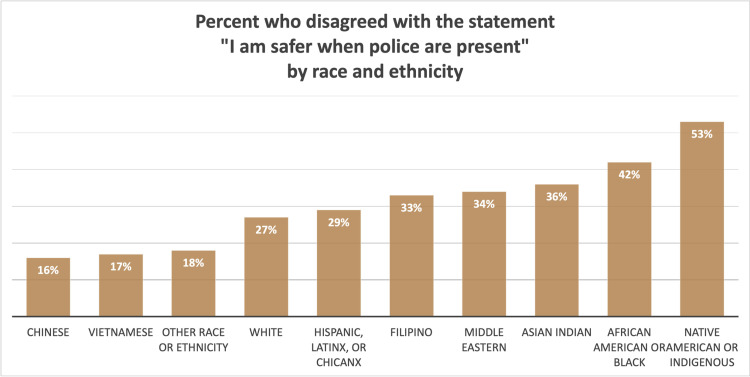
Percent who disagreed with the statement “I am safer when police are present” by race and ethnicity.

Each of the three statements about people’s sense of safety and responsiveness of the police had differences by demographic group, which were patterned in a consistent manner across gender, sexual orientation, race and ethnicity, income, nativity, employment status, and education level. Fig 6 illustrates this patterning by gender and sexual orientation: the groups most likely to disagree with the statement “I am safer when police are present” were those who identified their sexual orientation as Queer (75%) or their gender identity as non-binary, transgender or two-spirit (66%). The groups least likely to disagree with the statement identified as heterosexual men (20%) or heterosexual women (22%). Fig 7 illustrates this patterning by race and ethnicity, excluding groups with fewer than 20 people: the groups most likely to disagree with the statement “I am safer when police are present” were those who identified as Native American or Indigenous (53%) or African American or Black (42%). The groups least likely to disagree with the statement identified as Chinese (16%) or Vietnamese (17%). People in households earning more than $200,000 per year were much more likely to report feeling safer when police are present than people with lower household incomes. Those born outside the United States were more likely to report feeling safer when police are present than people born in the United States. Considering employment status, retired people were the most likely to report feeling safer when police are present and people on disability were the most likely to disagree with this statement.

We conducted an intersectional analysis to examine the interaction of several identity markers on the question of whether people feel safer when police are present. Participants who were white, heterosexual, cisgender men or women, with household incomes greater than $100,000 per year were more than twice as likely to report that they felt safer when police are present than participants who were non-white, lesbian, gay, bisexual, transgender, non-binary, or queer, with household incomes below $100,000 per year (Prevalence Ratio: 2.27, 95% CI 1.56, 3.30, *p* < 0.001).

### Direct experiences with police

**Fig 8:**
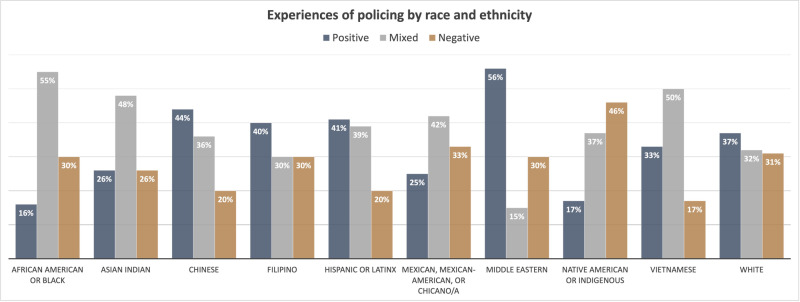
Experiences of policing by race and ethnicity.

The survey also asked people to reflect on any experiences that they may have had with the police in the past five years and to report whether those experiences were generally positive, mixed, or negative. Just over half (51%) of the survey respondents had experiences with the police in the previous five years with 37% generally positive, 36% mixed, and 27% negative. Among the group who had prior experience with the police in the previous 5 years, negative experiences were more common for people who were unemployed or disabled compared to other employment statuses. People who identified as straight had more positive experiences compared to lesbian, gay, or bisexual participants. Men reported more positive experiences (45%) compared to women (33%), which was more than twice the level of positive experiences reported by non-binary, transgender, or two-spirit participants. There were also large differences in experience by race and ethnicity. Fig 8 describes the experiences of respondents by racial and ethnic groups, excluding groups with fewer than 20 people. In an intersectional analysis, among those who had an experience with the police in the previous five years, those who were white, heterosexual, cisgender men or women, with household incomes greater than $100,000 per year were almost three times as likely to report a positive experience compared to those who were non-white, lesbian, gay, bisexual, transgender, non-binary, or queer, with household incomes below $100,000 per year (Prevalence Ratio 2.89, 95% CI 1.55, 5.37, *p* < 0.001).

### Perspectives on alternatives to policing

The survey assessed participants’ support for four specific initiatives to shift certain responsibilities away from the police and toward civilian agencies. These initiatives were being considered or piloted in the region and reflected four common domains where police are often involved and public policy was being considered to shift responsibility to civilian actors [37]. The four initiatives were:

1. Develop a mental health crisis team that responds to emergency (911) calls for some types of mental health or addiction problems instead of the police (for example, where the caller does not think there is risk of violence).
2. Invest in better bike lanes, lighting, and crosswalks, and automated tools for enforcement of traffic laws (like broken tail lights or expired registration) rather than police stops.
3. Invest in meeting the shelter, medical, and basic needs of homeless populations instead of evicting people from encampments or charging homeless people with loitering.
4. Increase the number of trained counselors and coaches in San José schools to replace police School Resource Officers.

**Fig. 9:**
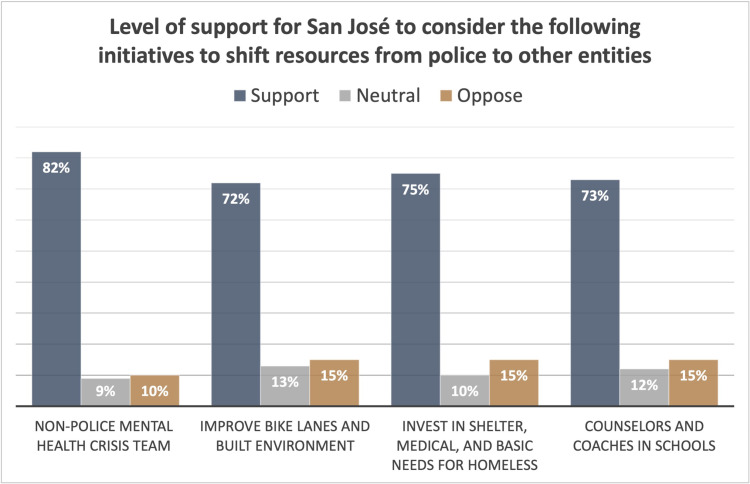
Level of support for San José to consider the following initiatives to shift resources from police to other entities.

Each of the four initiatives had a strong degree of support, with 82% of respondents supporting initiative 1 and between 72–75% of respondents supporting initiatives 2, 3, and 4 (Fig 9). In addition, in a separate question, 58% of respondents expressed that they felt that community safety would be better achieved through spending less money on policing and more on education, health care, and housing. We explored whether the level of support was different by gender, sexual orientation, race and ethnicity, income level, age, and nativity. There were only slight differences between groups and all groups had a strong majority supporting each of the four alternatives to policing.

## Discussion

In this city-wide assessment, we found that San José residents were generally supportive of increasing funding for community safety resources, helping residents meet their basic needs, and investing in public resources like parks, libraries, and transportation. Across demographic categories, respondents supported San José adopting alternatives to policing with between 72% − 82% supporting non-police approaches to managing mental health crises, traffic safety, school safety, and the needs of the city’s unhoused population. The survey found broad support among San José residents for several of the policy proposals put forth by Silicon Valley De-Bug in their Protect Your People Budget, including funding community-based alternatives to police responding to behavioral health crises [26].

Nearly half the participants supported a reduction in funding for police. The level of support for shifting spending and responsibility away from police among San José residents was higher than documented in national opinion surveys conducted by the Pew Research Center in 2020 and 2021 [8]. In 2020, 25% of adults in the survey supported reducing spending on police and in 2021, 15% of adults supported decreased spending [8]. However, a 2022 opinion poll of California voters tells a story that is more consistent with our survey responses: respondents thought that the most common situations in need of emergency response in their community were crises related to homelessness (49%) and mental health (38%) and they favored behavioral health specialists responding to these types of emergencies (69%) either with police (35%) or independent of a police response (34%) [11].

Consistent with the extant literature, residents’ experiences with police varied widely based on their demographic groups [12]. Across a range of questions, sexual and gender minorities, younger people, respondents who identified as African American or Black, Native American, or Chicanx/Latinx/Hispanic, and people with lower household incomes had generally more negative experiences with and attitudes towards policing compared to those who identify as men, heterosexual, older, White or Asian, or had higher household incomes. In our intersectional analyses, considering the effect of occupying multiple intersecting marginalized identities, we found that participants who were white, heterosexual, cisgender men and women, who earn more than $100,000 per year were more likely to report that they felt safer when police were present, and of those who had experiences with the police in the previous five years, these were more likely to be positive experiences, compared to participants who identify as non-white, lesbian, gay, bisexual, transgender, non-binary, or queer, and earned less than $100,000 per year.

While these differences are essential to document to support understanding of diverse community members’ experiences with policing, it is notable that support for alternatives to policing and for shifting resources away from police and towards other means of achieving community safety was robust across demographic groups.

Our findings are similar to the results from the “Policing the Rainbow” study, a nationally representative survey of nearly 1,500 adults that oversampled LGBTQ+ individuals [15]. The study found that LGBTQ+ people are more likely to be crime victims but less likely to report these events to the police. They also identified significant differences by race, gender identity, sexual orientation, and socioeconomic status within the LGBTQ+ population: people of color, and those who identify as transgender, nonbinary, bisexual and queer reported more mistreatment by police and more reluctance to use police than those who identify as white LGBTQ+ people, cisgender, and gay and lesbian. The study found that Asian LGBTQ+ people reported the highest trust in police and had the lowest level of police-initiated contact, such as being stopped, searched or held in custody.

There have been few surveys broadly assessing people’s perspectives on spending for community safety. In the summer of 2020, the People’s Budget Los Angeles Coalition engaged in participatory budgeting through interactive online engagements and a survey of over 24,000 residents [18]. Participants reported high priority areas for investment in housing, public health and healthcare, child and youth development, food security, public transportation, and environmental justice. Priority areas for divestment included parking enforcement, police and policing, and the City Attorney’s Office.

The national Latinx organization, Mijente, commissioned a large, representative survey of Latinx adults to assess perspectives on community safety, policing, and the Black Lives Matter movement in 2021–2022 [16]. There were 1,359 participants and political perspectives varied dramatically, reflecting the diversity of the Latinx population in America [16]. A majority of respondents reported that they or a close family member had had a negative encounter with police, with these experiences racially patterned. For example, 44% of Afro-Latinx participants reported a police officer had been aggressive with them, compared to 19% of Latinx participants who identified as white. Similarly, the survey found that 41% of Afro-Latinx respondents had avoided calling the police for fear the police might make the situation worse, almost double the number of those who identify as white (22%) [16]. Over 80% of Latinx respondents supported mental health response rather than law enforcement for mental health crises [16]. Similarly robust majorities in this survey stated that access to affordable quality housing (72%), childcare (67%), healthcare (71%), strong neighborhood ties (69%), and parks (60%) would “help a lot” to improve community safety, compared to a minority who thought police patrols would “help a lot” (46%) [16].

As illustrated by the diversity of perspectives, experiences, and opinions reflected in the present study, the Policing the Rainbow study, and the study commissioned by Mijente of Latinx Americans, we caution against simplification of any community’s perspectives and experiences based on either identity or geography. The diversity of political ideologies, races, and experiences within the Latinx respondents in the Mijente study reflects the diversity of the Latinx population within the United States. Similarly, in a qualitative study of residents’ comments about community safety in Oakland, CA, just north of San José, Levy, Lerman, and Dixon found several complex and contested ideas of what “community” meant and cautioned against conflating geographic location with “community,” noting that “the simple fact of sharing space does not guarantee the presence of a shared sense of community between residents” [38].

### Limitations

While the findings from this survey are robust, we would like to note some limitations. This study presents the results of a survey of residents of one city and the specific findings should not be generalized beyond San José. We believe the methods we present here can be replicated in other communities. While we had a large, diverse sample of participants, indeed larger than many other surveys of experiences of policing, the sample was not designed to be representative. We employed multiple survey solicitation approaches, including texting voter registration rolls, tabling at in person events like music festivals, and email announcements made by a diverse range of community-based organizations and city officials. While several of the community-based organizations have a more progressive political orientation, other organizational partners, such as health service agencies and non-profits to support local parks, do not. The majority of survey responses were collected via the mass texting activities. While our sample broadly reflects San José’s racial and ethnic distribution, our sample is slightly more educated and has a lower proportion of residents who were born outside the United States than the general population of San José. It is reasonable to assume that there was a form of volunteer/non-response bias, where people who are motivated by the issues of community safety and policing were more likely to participate than people who care less about these issues. It is also possible that there was a priming effect in asking questions about experiences of policing and opinions about policing prior to asking about support for alternatives to policing.

In addition, though we had gender-diverse participants, the wording of our question ascertaining gender identity meant that we were unable to distinguish transgender women from other groups. This is unfortunate as research has shown transgender women often have negative experience with policing and transgender women of color, in particular, report significantly more assaults by police officers than other groups [39]. In addition, we collected insufficient information on disability status due to the addition of the question on disability status late in the survey. Having a disability has also been shown to be associated with negative experiences of policing [39].

## Conclusion

The purpose of this study was to develop a deeper understanding of the perspectives of San José residents on community safety, policing, and the city budget. Developed collaboratively with representatives from dozens of local organizations and offered in seven languages, the survey engaged a diverse sample of residents and yielded actionable information to support policy development, coalition building, and organizing [31]. While the specific results of this study may not be generalizable beyond the San José community, these results do add complexity and nuance to a complex topic, and suggest intersectional methods are feasible and appropriate. While San José residents had widely differing experiences with police, there was broad agreement on policy proposals to support alternatives to policing.

Many policy decisions are inherently local, and can be supported by community-based participatory research strategies that engage the public [1,37]. As communities across the country grapple with how to achieve safety for all residents, including those who bear the disproportionate harm from policing, community-based participatory research strategies can be a valuable tool for engaging, understanding, and mobilizing communities [4].

## Supporting information

S1 Supplementary Table San José Community Safety Survey. (DOCX)
