# Supplementary material for: Diverse residents’ experiences of policing and attitudes towards law enforcement: Findings from a large community-based survey in San José, California
Source: PLoS One. 2025 May 29;20(5):e0325257. doi: 10.1371/journal.pone.0325257 (PMC12121803; doi:10.1371/journal.pone.0325257)
Supplement: S1 Supplementary Table — (DOCX) [file pone.0325257.s001.docx]

**S1 Supplementary Table. San José Community Safety Survey.**

| Do you live in San José? |
| --- |
| Yes  No |
| What is your zip code? (open response) |
| Please indicate whether you “agree” or “disagree” with the following statements. If undecided, please select “neutral.” |
| 1. I am safer when police are present. 2. Our community would be safer if we spent less taxpayer money on policing and more on education, health care, and housing. 3. People should be jailed for non-violent crimes. 4. Police should have access to military-grade weapons (like tanks, grenade launchers, and .50+ caliber weapons). 5. I hesitate to call the police for help. 6. Cities (taxpayers) should pay the full price of lawsuits when police officers and departments are sued in civil court, relieving the officer of financial responsibility. 7. When I need help from the police, they respond in a timely and appropriate manner. |
| Which of these statements best describes your opinion? |
| - Policing in San José is working well and does not need reform. - Policing in San José has some problems, but they are caused by individual bad actors, so major reforms are not necessary. - Policing in San José has some serious problems, requiring major reform and shifting some resources to other approaches to creating public safety. - I don’t know. |
| Motivated by social protest about police violence, cities across the country are evaluating whether to shift certain responsibilities away from the police and toward civilian agencies. Please share your level of support for San José to consider the following initiatives: [RESPONSE OPTIONS: Strongly oppose, somewhat oppose, neutral, somewhat support, strongly support] |
| 1. Develop a mental health crisis team that responds to emergency (911) calls for some types of mental health or addiction problems instead of the police (for example, where the caller does not think there is risk of violence). 2. Invest in better bike lanes, lighting, and crosswalks, and automated tools for enforcement of traffic laws (like broken tail lights or expired registration) rather than police stops. 3. Invest in meeting the shelter, medical, and basic needs of homeless populations instead of evicting people from encampments or charging homeless people with loitering. 4. Increase the number of trained counselors and coaches in San José schools to replace police School Resource Officers. |
| Should the city of San José spend more, less or the same amount of money on the following services in coming years?  [RESPONSE OPTIONS: Spend less money, spend the same amount of money, spend more money] |
| 1. Helping residents meet basic needs (food, housing, economic assistance, etc.) 2. Community safety resources  (community care workers, community clinics, restorative justice programs, programs for elders and youth, etc.) 3. Public resources like parks, libraries, or transportation 4. Policing |
| Do you have any suggestions about what San José’s budget priorities should be? (open response) |
| Have you had any experiences with police or law enforcement in the past 5 years? |
| No  Yes  Don’t know  Prefer not to answer |
| [IF YES IS SELECTED, THEN THE FOLLOWING QUESTION IS ASKED]  Would you say your experience was generally positive, negative or mixed?  Positive  Negative  Mixed |
| What is your age? |
| 12 or younger  13-17  18 - 25  26 - 39  40 - 64  65 or older |
| What is your gender? |
| Man  Woman  Non-binary or gender non-conforming  Transgender  Two-Spirit  Some other gender identity |
| How would you describe your sexual orientation? |
| Straight/Heterosexual  Bisexual  Gay  Lesbian  Queer  Two-Spirit  Some other sexual orientation |
| What is your race or ethnicity (select all that apply) |
| African American or Black  Asian Indian  Chinese  Filipino  Japanese  Korean  Vietnamese  Other Asian  Chicano/a, Mexican-American, or Mexican  Hispanic or Latinx  Middle Eastern  Native American or Indigenous  Pacific Islander or Native Hawaiian  White  Some other race or ethnicity |
| What is the highest level of education you have completed? |
| Less than high school  High school degree  Associate’s degree  Bachelor’s degree  Graduate/Professional degree  Other |
| What is your total household income? |
| Less than $24,999  $25,000 to $49,999  $50,000 to $99,999  $100,000 to $199,999  $200,000 or more |
| Were you born in the United States? |
| Yes  No  I do not wish to answer |
| Have you or a member of your household spent time in jail, prison, or immigration detention in the last six months? |
| Yes  No  I’m not sure  I do not wish to answer |
| Are you currently experiencing homelessness/houselessness? (e.g. couch surfing, living in a motel, car or tent, or unsheltered)  Yes  No |
| What is your employment status? I am currently… |
| Working full time (40 hours or more)  Working part time (less than 40 hours per week)  Student  Unemployed  Disabled  Retired  Other |
| What languages are spoken in your home? (Select all that apply) |
| Spanish  Vietnamese  Chinese (Mandarin, Cantonese)  Tagalog (Filipino)  Hindi  Persian (Farsi, Dari)  Korean  Tamil  Telugu  Amharic, Somali, or Other Afro-Asiatic Languages  Punjabi  Japanese  Arabic  Russian  Ilocano, Samoan, Hawaiian, or Other Austronesian Languages  Nepali, Marathi, or Other Indic Languages  Gujarati  Portuguese  Malayalam, Kannada, or Other Dravidian Languages  French  Urdu  Serbo-Croatian  German  Khmer  Greek  Bengali  Other (Please specify) |
| Do you have a disability or health condition that impairs your physical, cognitive, neurological, behavioral, emotional, learning, hearing, or visual function? |
| Yes  Sometimes  No |
| [IF YES OR SOMETIMES IS SELECTED ABOVE, THEN ASK:]  Mark below any area of disability or health condition you have that could impair your ability to interact with police or law enforcement. (Select all that apply)  Physical movement  Hearing/vision  Cognition/learning  Neurological  Behavioral  Emotional  Some other condition  I do not have a disability that could impair my ability to interact with police or law enforcement |
